# Exsection of a Portion of the Right Malar Bone for Removal of Necrosis

**Published:** 1863-01

**Authors:** T. T. Lockwood

**Affiliations:** Buffalo, N. Y.


ART. VI.—*Exsection of a portion of the right malar bone for removal of Necrosis.* *By* T. T. LOCKWOOD, M. D., *Buffalo, N. Y.*

Anton Brizolaro, by birth a Russian, aged 10 years, of healthy parents. When six years old received a severe blow upon the face, the force being received mainly upon the maxillary process of the malar bone. Soon after the injury, the first effects of which were never fully recovered from, the face commenced to swell and inflame, and became very painful; ulcerative action soon produced abundant discharge from an opening on the middle portion of the bone; this had continued in a greater or less degree from that time until the operation for removal of the portion of bone which had been diseased. Various unsuccessful attempts for relief had been made both by physicians in private practice, and also in the hospitals he had visited with the view of removal or partial relief from the attending discharge and constitutional depression, but probably the necrosed portion had not sufficiently separated from the healthy at that time to make removal practicable without cutting it entirely from its attachment, and possibly the extent of disease or the line of demarkation could not be satisfactorily ascertained.

November 1st.—Incision was made down upon the bone along the orbital edge, nearly its whole length, and the diseased portion fully exposed.

After ascertaining its extent and attachments, with strong forceps, the necrosed portion was removed nearly entire, and after smoothing the surface and carefully cutting away all roughened and diseased parts, the integuments were approximated and simple dressings applied. In about six weeks the wound had completely healed, and the general health of the lad greatly improved.

The cicatrix constitutes no great deformity, though the lower eye-lid is slightly depressed, and a cup-like depression remains where the integuments at the base, rest very closely upon the bone. The integuments and bone may be said to have healed by first intention, and this may be considered one of the important features of the case; indeed the interest chiefly attached to it, and the object of presenting it for publication, consists in the rapidity with which the wound healed, when bone structure had been so long involved in disease, and also the great and almost instantaneous improvement which followed in the general health, which had severely suffered for so long period, when the discharge was arrested and the system relieved from this constant source of depression and irritation.

